# Supplementary material for: Ghost lineages can invalidate or even reverse findings regarding gene flow
Source: PLoS Biol. 2022 Sep 14;20(9):e3001776. doi: 10.1371/journal.pbio.3001776 (PMC9473628; doi:10.1371/journal.pbio.3001776)
Supplement: S1 Material — (PDF) [file pbio.3001776.s001.pdf]

S1 Material. Additional simulations for exploring the effect of ghosts on the method employed by Fontaine et al. (2015) [1] to find the correct branching order of *Anopheles* species.

To explore the effect of ghost lineages on the observed divergence times in gene trees following or not the species tree topology, but without choosing arbitrarily a specific scenario as Fig 4 (main text), we used the approach detailed hereafter, using the software Zombi [2]:

- We simulated a species tree containing extinct (ghost) species. This tree was obtained using the **Tp** mode of Zombi, with a speciation and extinction rates of 0.15 and 0.09 respectively. The lineage number was set to 20 species for the 140 first units of times then to 3 species for the last 10 units with a turnover set to 0.05. This produced a complete tree of 119 species of which only 10 were extant (S1B Fig). These extant species were grouped arbitrarily in three clades, A, B and C, with topology ((A,B),C).
- We produced a new version of this species tree that did not contain ghosts by simply pruning them out (S1A Fig).
- On each version of the tree, we simulated the evolution of a genome evolving along its branches, letting interspecies introgression occur. Genomes with an initial size of 3000 genes were made to evolve in both trees using the **G** mode in Zombi. Introgression was simulated by setting the transfer rate of genes to 5 for the tree with extinct species and 10 for the other one. The probability of replacement transfers was set to 1. The extension parameter of the transfer events was set to 1, meaning that every time that a transfer event occured it affected only a single gene.
- We recovered the gene trees at the end of the simulations and we grouped them according to which topologies they supported: either the topology reflecting the species history: ((A,B),C) or one of the two topologies resulting from introgressions, ((A,C),B) and ((B,C),A). For each of the three topologies and for each simulation, mean T1 and T2 were computed
- The results (S1 Fig) were presented following Fig 3 (A and C) of [1].

References

1. Fontaine MC, Pease JB, Steele A, Waterhouse RM, Neafsey DE, Sharakhov IV, et al. Extensive introgression in a malaria vector species complex revealed by phylogenomics.

Science. 2015;347: 1258524. doi:10.1126/science.1258524

2. Davín AA, Tricou T, Tannier E, de Vienne DM, Szöllősi GJ. Zombi: a phylogenetic simulator of trees, genomes and sequences that accounts for dead linages. Schwartz R, editor. Bioinformatics. 2020;36: 1286–1288. doi:10.1093/bioinformatics/btz710
